# In Vivo Production of RNA Aptamers and Nanoparticles: Problems and Prospects

**DOI:** 10.3390/molecules26051422

**Published:** 2021-03-06

**Authors:** Ousama Al Shanaa, Andrey Rumyantsev, Elena Sambuk, Marina Padkina

**Affiliations:** 1 Department of Genetics and Biotechnology, Saint Petersburg State University, 199034 Saint Petersburg, Russia; a.m.rumyantsev@spbu.ru (A.R.); e.sambuk@spbu.ru (E.S.); 2 Atomic Energy Commission of Syria, Damascus P.O.B 6091, Syria

**Keywords:** aptamer, nanoparticles, nucleic acids, nucleic acid nanoparticles

## Abstract

RNA aptamers are becoming increasingly attractive due to their superior properties. This review discusses the early stages of aptamer research, the main developments in this area, and the latest technologies being developed. The review also highlights the advantages of RNA aptamers in comparison to antibodies, considering the great potential of RNA aptamers and their applications in the near future. In addition, it is shown how RNA aptamers can form endless 3-D structures, giving rise to various structural and functional possibilities. Special attention is paid to the Mango, Spinach and Broccoli fluorescent RNA aptamers, and the advantages of split RNA aptamers are discussed. The review focuses on the importance of creating a platform for the synthesis of RNA nanoparticles in vivo and examines yeast, namely *Saccharomyces cerevisiae*, as a potential model organism for the production of RNA nanoparticles on a large scale.

## 1. Introduction

Aptamers are single-stranded DNA or RNA oligonucleotides, characterized by their various 3-D conformations, resulting in a distinctive ability to recognize and bind to numerous targets with high specificity. Such targets include metal ions, nucleic acids, proteins, polysaccharides, and other organic compounds, in addition to viruses, subcellular organelles, and cells [1,2,3,4,5,6,7,8]. The term “aptamer” was first coined by Szostak and Ellington [9], who blended two words of Latin and Greek origin, namely aptus—fit—and meros—part—and together they mean fitting parts in English. With an estimated market value of USD billion by 2025, aptamer research is a highly dynamic interdisciplinary field of science and technology [10].

The first nucleic acid-based aptamers were concurrently produced in both Gold’s and Szostak’s laboratories in 1990 using SELEX (systematic evolution of ligands by exponential enrichment). This technology depends on a library of random single-stranded DNA or RNA molecules, the sequences of which are flanked by identical sequences at both 3′ and 5′ ends [9].

SELEX is designed to enrich the oligonucleotides with high affinity to the target molecules by means of three main steps for each cycle: A) the oligonucleotides from the library bind to their specific targets, B) the unbound oligonucleotides are eliminated, and C) the bound oligonucleotides are amplified using PCR using the common 3′ and 5′ flanking sequences. After the enrichment step, the oligonucleotide libraries showing the desired results are selected and the sequences are synthesized in sufficient quantities in order to carry out functional tests [11].

Methods of automated selection of aptamers have been developed in the interest of simultaneously determining aptamers for a number of different targets [12]. In silico ethods are becoming increasingly popular in the selection of aptamers due to their cost and time-effectiveness. The computational methods heavily rely on molecular docking and simulation tools, in which large numbers of aptamers can be screened against a certain ligand. Assisted by trial and error, and site-directed mutagenesis, the aptamers showing the highest affinity for the target particle can be synthesized and examined in vitro and in vivo (major in silico methods are reviewed extensively by Sabri et al., 2019 [13]. Popular methods include CLADE (closed loop aptameric directed evolution), where oligonucleotide sequences, randomly developed in silico or selected in vitro for their ability to interact with target ligands, are synthesized on microarrays. The most suitable sequences are then determined in vitro after analyzing the intensity of binding to the target ligands. Using directed evolution algorithms, mutations are introduced into oligonucleotides and a next-generation library is obtained. The cycle continues until the most effective aptamers are identified. This method has been successfully employed for designing DNA aptamers against the light-harvesting protein allophycocyanin, found in cyanobacteria and red algae [14]. 

Other promising in silico tools are making the most of the advances in artificial intelligence and machine learning. A number of tools for RNA aptamer clustering, motif finding, and aptamer optimization are discussed by Hamada, 2018 [15]. For RNA riboswitches, machine learning platforms such as SMOTE, Python 3, and WEKA3.8 have been reported by Beyene et al., 2020 [16] in order to statistically determine the desired sequence motifs, especially in undersampling cases to overcome data imbalances.

Nowadays, aptamers have high rates of demand in both fundamental and applied research. Aptamers with a reporter group are successful tools for the identification and localization of different molecules in cells, hence evaluating their roles in certain biological processes [17,18].

Aptamers can also help to identify specific sequences of nucleic acid-binding proteins and give rise to affinity reagents for protein purification [19,20,21,22]. Moreover, aptamers can also regulate gene expression as competitive inhibitors of transcription factors [23].

Another application of aptamers is in being the sensitive element of various biosensors for biomedical and environmental applications. Microorganisms can be detected using aptamer-based biosensors, including some aptamers designed to target HBV, HCV, SARS-CoV, Vaccinia, and Ebolavirus, reviewed by Zou et al. [6]. Aptamers against a number of bacterial pathogens have also been reported by Alizadeh et al. [24] and they can be used for both treatment and diagnosis. This new approach provides an alternative to the conventional anti-infective agents and helps to solve the problem of antimicrobial resistance.

As potential therapeutic agents, DNA aptamers have been shown to effectively bind and inhibit thrombin, offering a promising anti-coagulant after optimizing some pharmacokinetic properties [25]. Aptamers with reporter groups have wide applications in diagnostics, tumor localization, and the detection of target proteins with certain pathological relevance, all traditionally performed using antibodies [17,26,27].

Due to the high affinity and specificity of aptamers after many rounds of selection, in addition to their superior properties regarding shelf life, the ability to restore the 3-D structure after denaturation, low molecular size, low immunotoxicity, short time of development, and, more importantly, the wide range of targets, aptamers could replace antibodies at any point in the foreseeable future [28,29]. Accordingly, aptamers are often called chemical antibodies, lacking the disadvantages of classic immunoglobulins [30]. Antibodies against a certain substance are firstly produced in animals. The substance is introduced to the animals to generate an immune response—immunization. However, not all compounds are immunogenic, requiring high-cost and laborious methods to obtain suitable antibodies [30,31,32]. In contrast, aptamers can practically be developed for all sorts of chemicals, ranging from single ions to microorganisms [6,7,33,34]. Additionally, aptamers are significantly more stable than antibodies, even in the case of denaturation—unlike antibodies, they can restore the 3-D structure [28].

## 2. Functional and Structural Diversity of RNA: The Basis for Aptamer Development

In the past few years, awareness of the importance of RNA has steadily increased amongst researchers, owing to the ever-growing list of various RNA functions (reviewed by Breaker and Joyce) [35]. There is a large number of non-coding RNA (ncRNA) molecules in both prokaryotic and eukaryotic cells, mostly involved in many known biological processes, e.g., transcription, RNA maturation, translation, and epigenetic control of gene expression. Moreover, messenger RNA (mRNA) delivers the genetic information from the genome to the cell translational machinery. The highly structured ribosomal RNA (rRNA) and transfer RNA (tRNA) play a crucial role in translation. The former is the structural and catalytic part of the ribosome [36], and the latter is the adapter molecule [37]. Both synthetic and native ribozymes exhibit enzymatic activity [38,39,40]. Bacterial small RNA (sRNA) [41]; micro RNA (miRNA) [42]; and eukaryotic small interfering RNA (siRNA) [43] take part in the regulation of gene expression. Small nuclear RNA (snRNA) is involved in eukaryotic mRNA splicing [44]. Small nucleolar RNA (snoRNA) are involved in the chemical modification (methylation and pseudouridylation) and maturation of eukaryotic rRNA [45].

In fact, RNA aptamers selectively bind to various targets and influence their functions [46]. Aptazymes are artificial molecules made of an aptamer and a ribozyme, first introduced in 1997 [47]. Hence, aptazymes have two main structural motifs, one for recognition while the other is catalytic, in addition to a spacer region in order to control the intermolecular forces and interactions between the two motifs. Riboswitches are another example including an RNA aptamer motif and an expression motif. Upon ligand binding, the aptamer part of riboswitches induces conformational changes in the entire molecule, resulting in significant changes in the rate of translation and gene expression [48].

The functional diversity of RNA molecules unveils the huge potential for the development of new RNA-based drugs. The main candidates of such molecules fall into the groups of RNA aptamers, miRNAs, and siRNAs. The latter two molecules regulate and suppress protein synthesis, therefore making great antineoplastic drug candidates [49,50]. RNA aptamers, together with miRNAs and siRNAs, can greatly improve drug delivery to the target molecules.

One of the major drawbacks in embracing RNA for therapeutic purposes is the short half-life, due to the prevalence of nucleases in blood plasma. This pharmacokinetic parameter determining the distribution and clearance is of crucial importance for any drug. In fact, oligonucleotides exist for no longer than a few tens of minutes in blood plasma, rendering such drug candidates disadvantageous. However, this problem has been solved by introducing some chemical modifications to the nucleotides to protect the RNA polymer from the hydrolyzing effect of nucleases and by improving the pharmacokinetic properties while maintaining the pharmacodynamic activity. Another solution to prolong the half-life is by using RNA nanoparticles [51,52,53].

In the case of using chemically modified nucleotides, they can be added before or after SELEX. For the first option, nucleoside triphosphate can be used for aptamer synthesis after introducing one of the following modifications to the 2′ position of the ribose sugar: 2′ amino pyrimidine, 2′ fluoropyrimidine, 2′-*O*-methylpyrine, and 2′-*O*-methylpyrimidine. Aided by special RNA polymerases, e.g., T7 RNA polymerase, 2-*O*-methylated pyrimidine monomers can be catalyzed to synthesize RNA polymers [54]. For the second option, it should be noted that adding any moiety can affect the conformation of the aptamer and its activity, accordingly. For this reason, modifications after SELEX are normally performed on 5′ and 3′ ends of the nucleotides by adding polyethylene glycol (PEG), biotin, or lipid components. To stabilize the aptamer, an additional bond is created between the 2′ oxygen and 4′ carbon in the ribose molecule, forming what is called “locked nucleic acid” (LNA) [53,55,56].

As a matter of fact, the 3-D structure and the function of any aptamer are interconnected. Some RNA structural motifs include open and stacked three-way junctions [57], four-way junctions similar to Holliday’s structures [58], kissing loops, 90° kinks [59], pseudo-torsional angles [60], and many other structures. Virtually, an unlimited number of different structures can be designed out of the aforementioned motifs, enabling the aptamer to bind to all sorts of targets.

To create an aptamer and support its structure, it should be embedded in the supramolecular scaffold of an RNA molecule. Human tRNA^Lys^ is one of the well-studied RNA scaffold models, in addition to other tRNAs [61]. Embedding the aptamer in the anticodon stem within tRNA promotes proper folding. Additionally, this approach allowed heterologous synthesis of aptamers in bacterial cells, in amounts sufficient for conducting biochemical and crystallization experiments [62].

Resistance to intracellular nucleases is considered one of the most important criteria in the process of selecting supramolecular scaffolds to include the aptamer. The cleavage of the RNA scaffold can lead to the dissociation of the aptamer and loss of its action on the target. This is the disadvantage of tRNA-based scaffolds [63]. Employing 5S rRNA from *Vibrio proteolyticus* (V5) as an aptamer-bearing scaffold has also been reported, in which the helical domain III and loop C have been replaced by an aptamer. As a result, a functional aptamer against vascular endothelial growth factor (VEGF) has successfully been produced in *V. proteolyticus* (V5) [64].

More common scaffolds include phi9 3WJ, a stacked three-way junction (3WJ) motif from the bacteriophage phi29. This motif consists of three short RNA fragments (≤20 nucleotides); upon assembly, the overall structure shows high thermodynamic stability. It has been reported that this structure is stable in a solution containing 8 M of urea and it does not dissociate at lower concentrations [65].

The branching structure of Phi29 3WJ is very useful for inserting different functional modules into each of the three helices. This scaffold facilitates the correct folding of other molecules merged in its structure. Thus, this scaffold is capable of carrying different molecules, including aptamers, miRNAs, ribozymes, and even ligands that bind to cell receptors—each can be placed on a separate branch of the scaffold. Thanks to the correct folding of each molecule, their functions are maintained, including cell-binding, cell penetration, suppression of gene expression, catalytic functions, and others [66,67,68].

Nevertheless, in mammalian cells, along with the full-sized aptamers, based on Phi29 3WJ scaffold, some shortened variants of the aptamers have been found. One of the likely reasons for this issue is that near RNA-polymerase III terminator, there is a UUUGUU sequence, causing premature termination of transcription. Filonov et al. designed an F30 scaffold based on Phi29 3WJ after mutating the sequence, and premature termination discontinued [63].

## 3. Fluorescent RNA Aptamers

The variety of RNA functions in living cells have led to advanced method development to detect and study the dynamics of RNA in vivo [69].

For many years, the MS2-MCP method has been one of the most popular methods for RNA labeling, and it is based on the high-affinity binding of the bacteriophage coat protein MS2 (MCP) to the unique RNA hairpin sequence, the MS2 binding site (MBS). Therefore, cloning the MBS sequence into the RNA of interest and the simultaneous synthesis of MCP fused with the fluorescent protein GFP (green fluorescent protein) allows RNA localization in the cell [70,71].

However, background fluorescence from unbound MCP-GFP strongly affects the signal-to-noise ratio. In addition, it was found that the MS2 viral envelope proteins, associated with the MBS site in the 3’-untranslated region (UTR) of yeast mRNA, block the activity of Xrnp1 exonuclease and 5’-3’-degradation of mRNA. This leads to the accumulation of 3’ mRNA fragments that still bind to MCP-GFP, complicating in vivo full-length mRNA localization [72].

An alternative method for in situ RNA imaging is the use of fluorogenic RNA aptamers [73,74]. Since RNAs do not intrinsically show any fluorescence, an exogenous chromophore is required, the fluorescence of which is induced upon interaction with the RNA aptamer. Fluorogenic RNA aptamers are a powerful tool in RNA studies, and they are as good as GFP in protein studies. The insertion of a fluorogenic RNA aptamer into a target RNA molecule enables us to observe functioning RNA molecules in cells [75].

In 1999, Grate and Wilson proposed an RNA aptamer that binds to malachite green (MG) as a molecular biology tool [76]. The well-defined asymmetric loop in the RNA duplex provides high affinity and specificity of interaction with MG [77]. Laser radiation induces the hydrolysis of RNA at the MG binding site. As a result, inserting the nucleotide sequence of the aptamer into the target gene permits us to “mark” the obtained transcripts and leads to their destruction upon laser irradiation [76]. Both the degradation of the target RNA and the toxicity of MG and its derivatives to mammals and yeasts are the main disadvantages of fluorogenic MG aptamers [78].

In today’s RNA research, the most promising fluorescent RNA aptamers are the Mango, Spinach, and Broccoli.

## 4. RNA Mango Aptamer

The Mango RNA aptamer has an exceptionally high affinity to TO1-biotin, a thiazole orange derivative fluorophore, and upon binding, the fluorescence of the fluorophore is increased by 1100 times. The high affinity facilitates in situ low-copy RNA imaging, allowing the use of low concentrations of the fluorophore and thereby reducing the fluorescence background noise level and the adverse effect of the dye on cells [79]. The excitation and emission wavelengths of the Mango aptamer are 505 and 535 nm, respectively [80]. The Mango aptamer consists of 39 nucleotides and is one of the smallest fluorogenic RNA–dye complexes known to date [81].

**Figure 1:**
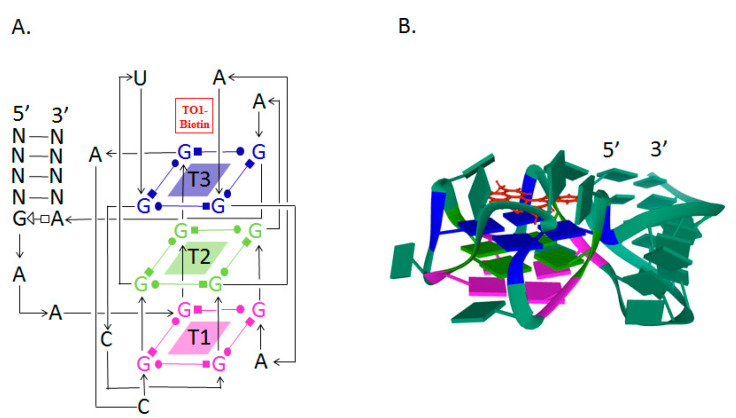
The secondary and tertiary structures of Mango RNA aptamer. (**A**) The secondary structure of Mango RNA aptamer showing the three G-quadruplex regions stacked in three tiers (T1, T2, and T3), where T3 serves as the binding site for TO1-biotin fluorophore [82,83]. (**B**) A cartoon representation of the tertiary structure of Mango RNA aptamer (PDB ID: 6C63) [84].

The analysis of the crystal structure of the Mango–TO1-biotin complex showed that the core of this aptamer, consisting of 23 nucleotides, is a three-level G-quadruplex (T1, T2, T3). TO1-biotin is adjacent to one of the nearly planar T3 faces of the G-quadruplex, and each of the three heterocycles of the fluorophore carries out stacking interactions with the nitrogenous bases of the RNA loop [82] (Figure 1).

Imaging of the Mango aptamer in *Caenorhabditis elegans* gonads using fluorescence microscopy demonstrates the potential of this system for studying RNA in living cells. The incorporation of the aptamer into bacterial 6S rRNA has provided a useful tool not only to label the molecule but also to purify it using affinity chromatography on streptavidin agarose [79].

Three new variants of Mango aptamers, I, II, III, with increased affinity, increased fluorescence, and resistance to salts and formaldehyde, were obtained by selection in the presence of a TO1-biotin competitive inhibitor. The latter circumstance allows the use of Mango aptamers not only in living cells but also in solutions ex vivo. Mango aptamers I, II, III folded with the F30 framework were successfully used for labeling and subsequent imaging of human 5S ribosomal RNA [83].

Increased levels of fluorescence can be achieved using RNA molecules with tandem repeats of the aptamer. It was shown that Mango II in triplex provides around 2.5 times higher fluorescence intensity than a single copy of the aptamer sequence. In this case, the localization of the target RNA —actin mRNA and NEAT1 long noncoding RNA—does not change [80].

## 5. Spinach and Broccoli Aptamers

Jaffrey et al. have synthesized several derivatives of 4-hydroxybenzylidene imidazolinone (HBI), which acts as a fluorophore in GFP reporter assay. Further, using SELEX technology, several aptamers were discovered that bind to the obtained fluorophores. The strongest fluorescence was demonstrated by 3,5-difluoro-4-hydroxybenzylidene imidazolinone (DFHBI) in the presence of a 98-nucleotide aptamer called Spinach [85]. Spinach enhances the fluorescence of the fluorophore by a factor of around 2000 [81]. The excitation and emission wavelengths of the Spinach aptamer were 452 and 496 nm, respectively. DFHBI does not induce cytotoxicity or phototoxicity. Aptamer insertion into 5S rRNA and its expression in mammalian cells have allowed the study of 5S rRNA distribution using fluorescence microscopy [85]. This indicates the cell permeability of the Spinach aptamer and the possibility of its application in RNA labeling and in vivo RNA imaging.

Chemical modifications of DFHBI, specifically adding a trifluoroethyl substituent to the methyl group (DFHBI-1T) or to the second carbon atom (DFHBI-2T), in the first case, result in an increase in the fluorescence intensity and, in the second, a shift in the excitation and emission maxima to the long-wavelength region of the spectrum. Thus, the fluorescence filters developed for YFP (yellow fluorescent protein) can be employed with DFHBI-2T [86].

Despite the above-mentioned advantages, the Spinach aptamer is not devoid of disadvantages, the main of which are thermal instability and a tendency towards improper folding at 37 °C, leading to a decrease in the fluorescence intensity. Using mutagenesis, Spinach2 aptamer was designed, which is more stable than Spinach, and it demonstrates the correct folding at 37 °C regardless of the fused RNA, particularly the 5S and 7SK RNA [87].

In addition, the use of tRNA as a scaffold increased the proportion of correctly folded aptamers of both types [61].

**Figure 2:**
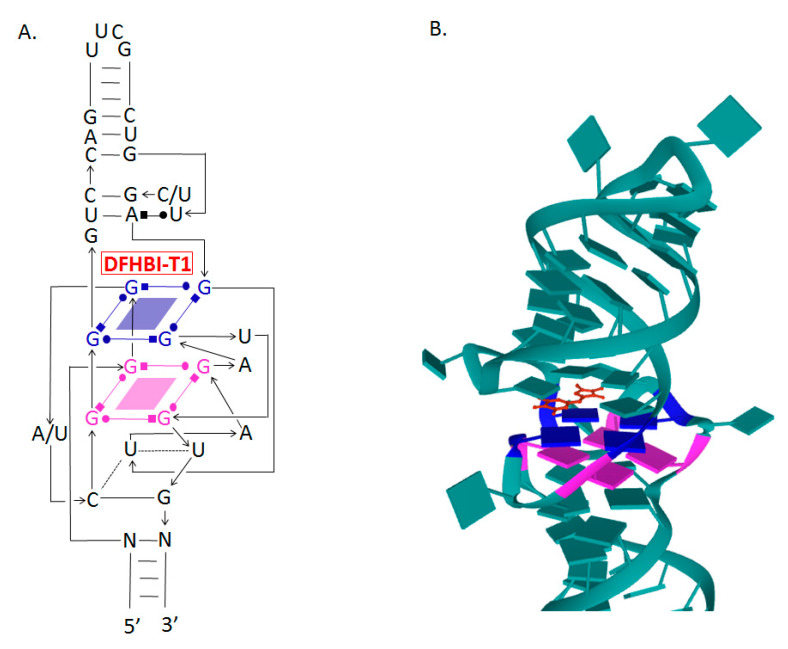
The secondary and tertiary structures of Broccoli and Spinach RNA aptamers. (**A**) The secondary structure of both aptamers showing the two G-quadruplex regions serving as the binding site to DFHBI-T1 [89]. (**B**) The tertiary structure of the fluorophore binding site in Spinach RNA aptamer (PDB ID: 6B14) [90].

Crystallographic analyses of Spinach aptamer structure showed that the RNA molecule has an elongated structure containing two helical domains separated by an internal loop. The loop folds into a G-quadruplex motif and it is flanked on both sides by antiparallel A-form duplexes. The G-quadruplex motif and the adjacent nucleotides form a partially formed fluorophore binding site. The intermolecular bonding between the fluorophore and the RNA aptamer is mediated by hydrogen bonds and π–π stacking interactions [88] (Figure 2).

Determining the molecular structure of Spinach and understanding the role of different regions in the sequence allowed the removal of parts of the sequence and the design of variants of the aptamer, e.g., Baby Spinach, which consists of 51 nucleotides only, with a fluorescence intensity comparable to that of the original variant [91].

To enhance the Spinach fluorescence signal and detect low-copy RNAs, tandem repeats of the aptamer were used. It was shown that 64 repeats of the aptamer increased the fluorescence intensity by 17 times [88].

Spinach aptamer has been used to create the sensitive element of biosensors designed for specific metabolites in bacterial cells. For this purpose, RNA sequences responsible for binding to certain metabolites were inserted into one of the stems of the Spinach aptamer. This modification of the aptamer structure led to our understanding that the correct structure of Spinach and the ability to interact with DFHBI were determined by the presence of these metabolites, and the level of fluorescence depended on their concentration [92]. Thus, the functions of the fluorogenic aptamer and the riboswitch have been combined in one molecule. Using such biosensors, the dynamics of the synthesis of ADP, S-adenosine methionine, guanine, and GTP in *Escherichia coli* was observed [93,94].

On the basis of Spinach, similar biosensors were also developed for monitoring proteins, namely thrombin, streptavidin, and the envelope protein of the MS2 phage [95].

The combination of SELEX and FACS (fluorescence-activated cell sorting) technologies has provided a powerful tool to develop a new version of the Spinach aptamer. This 49-nucleotide aptamer, named Broccoli, activates DFHBI or DFHBI-1T fluorescence, folds faster, shows high stability, and does not require a tRNA scaffold in vitro. The excitation and emission wavelengths of this aptamer are 472 and 507 nm, respectively [91]. The Broccoli aptamer has a higher affinity for fluorophores and the Broccoli–DFHBI-1T complex displays a brighter signal than Spinach–DFHBI [84]. Broccoli retains most of the G-quadruplex-forming nucleotides from the DFHBI-binding pocket in Spinach2 and probably has a similar structure upon interaction with DFHBI-1T [86,96,97] (Figure 2).

The secondary structure of Broccoli includes a hairpin-stem-loop and allows the production of aptamer dimers by replacing the terminal loop with a second aptamer molecule, leading to a 70% increase in fluorescence [96].

An additional advantage of Broccoli, like Spinach, is the ability to image the aptamers in vitro; fluorescence can be observed in microcentrifuge tubes [83] or by electrophoresis in polyacrylamide gel stained with DFHBI [64].

Both Spinach and Broccoli aptamers fused to the tRNA backbone have been successfully expressed in bacterial and mammalian cells. The ability of the Broccoli aptamer to fold in vitro without the aid of a tRNA scaffold has been confirmed in vivo. The RNA of the aptamer was fused to the 3’ end of 5S RNA and the resulting plasmids were transfected into HEK293T cells. Using flow cytometry, 5S RNA–Broccoli was detected in the cells, and the brightness of the cells was higher compared to cells containing 5S RNA–tRNA–aptamer. This supports the idea that the tRNA backbone is often cleaved by cell nucleases and, thus, has a negative effect on the expression of RNA aptamers. It should be noted that Spinach2 folding requires a tRNA backbone, and no fluorescence was detected when using the 5S RNA–Spinach2 construct [96].

Expression of the Broccoli–DFHBI-1T and Spinach–DFHBI aptamers in the 16S ribosomal RNA has allowed ribosomal imaging and provided a unique opportunity for studying translation in prokaryotes [98].

Broccoli–DFHBI-1T and Spinach–DFHBI were inserted into the 5’-hairpin of one of the yeast H/CA small nucleolar RNAs (snoR30), which is involved in rRNA maturation. The yeast cells were transformed with plasmids containing these constructs under the control of the GAL promoter. The growth of the transformants did not significantly differ from the growth of the parent line; aptamers did not disrupt the localization and function of snoR30 and provided fluorescence in the nucleoli [98].

The Spinach2–tRNA and Broccoli–F30 aptamers were used to study the regulation of RNA synthesis of the SINV virus, which can cause seasonal outbreaks of rash and arthritis in humans and encephalomyelitis in experimentally infected mice. Consequently, aptamers were inserted into the 3’UTR of viral RNA. The resulting recombinant viruses replicated well in nerve cells and BHK fibroblast cell culture. The fluorescence level correlated with the Broccoli–F30 copy number [99].

On the basis of the Broccoli aptamer, a fluorometric RNA substrate was developed, the fluorescence of which was proportional to the activity of RNA-modifying enzymes. Thus, a variant of the aptamer with modified nucleotides, such as N6-methyladenosine (m6A), was synthesized. Such an aptamer is unable to function normally, or restore its function, and special demethylases are required to restore its function. This approach facilitates the search for not only enzymes that modify RNA but also for their inhibitors and factors that affect the levels of RNA methylation in living cells [100].

New split RNA aptamers were developed to evaluate RNA co-transcriptional folding, RNA–RNA interaction dynamics, and RNA aptamer assembly in vivo. The split RNA aptamer consists of a pair of oligonucleotides that re-associate, when located in close proximity, and form the entire aptamer that can bind to the fluorophore and exhibit fluorogenic properties. A split Broccoli consisting of two strands of RNA—Broc and Coli—was developed, demonstrating high but incomplete complementarity. The dependence of the fluorescence of F30–Broccoli cleaved aptamers on temperature, the concentration of magnesium ions, and the presence of certain oligonucleotides allow these aptamers to be used as “molecular thermometers”, biosensors, and “molecular switches” [101,102,103].

Thus, aptamers can be used for biological imaging of nucleic acid and to study their dynamics in the cell and, therefore, studying the regulation of gene expression and metabolism. In addition, aptamers play important roles as biosensors for proteins and various other metabolites (see reviews by Dolgosheina, Unrau; Trachman et al.) [73,82]. RNA aptamers are used as a platform for creating effective antibacterial drugs that can independently inactivate bacterial cells and block the action of toxins secreted by pathogens, as well as other virulence factors [104]. Aptamers have wide medical applications in the diagnosis and treatment of diseases (see reviews by Asha et al.; Morita et al.; Dammes and Peer) [105,106,107].

Hybrid RNA–DNA molecules represent another variant of aptamers. The therapeutic potential of the RNA–DNA aptamer has been demonstrated for the treatment of melanoma. When these structures enter the cells, si-RNA and DS-DNA are released. si-RNA suppresses the mutated BRAF gene in melanoma cells. DS-DNA, which contains the binding site for NF-kB, holds it in the cytoplasm and blocks the activation of the NF-kB pathway, which increases the death of melanoma cells treated with vemurafenib [108].

A promising application of aptamers is targeted drug delivery, among which microRNAs and siRNAs are the most important. However, the bottleneck remains in effectively delivering the RNA to the target with minimal damage to healthy cells and tissues. These problems are partially solved thanks to nanoparticles [68,109].

## 6. Nucleic Acid Nanoparticles (Advantages of Nucleic Acid Nanoparticles)

The half-life of nanoparticles in the blood plasma, the target-oriented delivery, and the bioavailability of the active substances all depend on many parameters, particularly the composition, size, and shape of the nanoparticle and the method of ligand presentation. Various materials are used to obtain nanoparticles (see reviews by Hong et al.; Ni et al.; Lombardo et al.) [110,111,112]. The disadvantages of traditionally used liposomes and polymeric materials are the heterogeneity of the size, composition, and surface structure of the formed nanoparticles, which leads to a significant decrease in the efficiency of targeted delivery and uncontrolled localization of the ligands [51,113].

Peptides, proteins, and oligonucleotides such as DNA and RNA have significant potential for the formation of supramolecular functional nanostructures. Due to their physicochemical properties, these molecules allow the creation of nanostructures of a certain conformation to be used in various fields of biology, biotechnology, and medicine (see the review by Wang et al.) [8]. In recent decades, DNA and RNA oligonucleotides have become more popular as molecular building blocks for the creation of biocompatible nanostructures with controlled properties. The formation of these structures is based on canonical base interactions in RNA and DNA, as well as the possibility of additional non-canonical base interactions [114,115].

The DNA-based technology for producing nanoparticles called DNA origami [116] allows for better control of the composition of nanoparticles, which simplifies the preparation of practically identical nanostructures of various shapes with ligands that provide targeted delivery of the required compounds [117]. Special software based on the initial sequences and conformations of the DNA strands facilitates the assembly of nanostructures with a certain size, morphology, and functional groups that are necessary for the attachment of certain ligands [118].

For assembly of DNA nanoparticles, circular single-stranded DNA of bacteriophage M13mp18 is usually used (see review by Hong et al.) [119].

The application of DNA nanotechnology is especially promising in the field of nanomedicine and targeted drug delivery. To use DNA/RNA nanoparticles in medicine, it is necessary to study their efficacy, safety, and immunogenicity [120,121].

Research in recent years has demonstrated the potential of molecular engineering in the precise design of nucleic acid scaffold devices that are capable of performing complex tasks, such as targeting drug delivery and triggering a response in the cell or the entire body [122,123]. For siRNA delivery into HeLa cells, DNA-based nanoparticles were used, which have a tetrahedral shape with a height of around 7.5 nm. Each side of the tetrahedron consists of 30 nucleotides and contains an unpaired region, the protruding ends of which are complementary to the sequence of the siRNA. Thus, six molecules of siRNA can be fixed onto one nanoparticle [124].

Each nanoparticle also contains three folic acid molecules to bind to numerous folate receptors found in various tumors. Nanoparticles accumulating in tumor cells reduced the expression of pathologically associated genes by around two times. In the course of studies on mice with a xenograft of the human adenocarcinoma cell line KB, it was found that the blood circulation time of the siRNA in association with nanoparticles increased by six times; nanoparticles accumulated almost only in tumor cells and did not cause any significant immune response [119]. Recently, rectangular DNA particles with a size of 20–30 nm were developed from the DNA of the M13p18 bacteriophage for the direct delivery of the antitumor agent doxorubicin to ovarian tumor cells [125].

Despite the fact that the first nucleic acid-based nanoparticles were designed using DNA oligonucleotides, and these experiments served as the basis for the emergence of DNA origami technology, in recent years, RNA has become an increasingly important alternative in oligonucleotide nanotechnology [126,127].

Structural differences between DNA and RNA determine the differences in their properties. DNA is more chemically stable and has a higher melting point. Meanwhile, RNA is able to form more diverse 3-D structures than DNA [128].

The accumulation of knowledge about RNA in combination with the methods of synthesis and the selection of RNA molecules with certain properties has given rise to a new field of science—RNA nanotechnology [129]. RNA nanotechnology refers to the development, synthesis, and use of RNA nanoparticles, which, in most cases, are characterized by the ability to self-assemble and interact with other molecules [130].

Single-stranded oligonucleotides are the building blocks for creating complex and functional RNA nanostructures. The ability to devise nanoparticles with a specific structure and arrangement of functional groups opens up prospects for their use in biology and medicine [59,126].

## 7. The Need for a Framework for Developing Nanoparticles of Desired Shapes and Sizes

The secondary or tertiary structure of RNA and RNA nanoparticles can be predicted using in silico analysis of the nucleotide sequence [131,132]. The use of natural structural RNA motifs allows the assembly of nanoparticles [57,60,133,134].

The 3WJ motif of the “packing” RNA of bacteriophage phi29—Phi29 3WJ—is used as the central core for the production of RNA nanoparticles, as well as for aptamer folding (see the review by Guo et al.) [135].

The first RNA nanoparticles on the Phi29 3WJ platform were designed in 1998 [32]. The crystal structure of Phi29 3WJ has already been solved [136].

**Figure 3:**
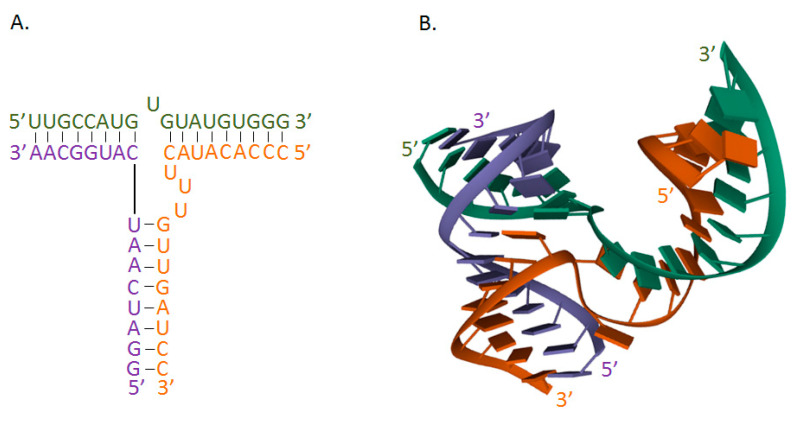
Structure of phi29 prohead RNA (pRNA) 3-way junction core. (**A**) The secondary structure of the pRNA. (**B**) The tertiary structure of the pRNA (PDB ID: 4KZ2) [137].

The branched structure of the 3WJ motif allows the facile integration of various functional modules into three helical sections (Figure 3). This “polyvalence” of the 3WJ motif facilitates the design of nanoparticles that can be used for the diagnostics and therapy of various diseases [57].

Structural elements of tRNA can be used as a framework for obtaining three-dimensional multifaceted nanoparticles. Such nanoparticles provide the intracellular and extracellular localization of ligands [138,139].

The integration of aptamers with RNA nanoparticles allows the targeting of a number of molecules, particularly cellular receptors, and aids in the active penetration of nanoparticles into cells through receptor-mediated endocytosis, or the passive penetration through the plasma membrane [140,141,142]. Lin et al. have extensively reviewed various types of RNA nanoparticles used for the treatment of cancer and immunomodulation [143].

Replacing RNA strands with either DNA or chemical analogs increases the enzymatic and thermodynamic stability of nanoparticles. It has been shown that nanoparticles of the same shape, size, and charge, but differing in chemical composition, show noticeable differences in their physicochemical properties, subcellular localization, and immunomodulatory effect. A composition of nanoparticles can be proposed that will provide targeted drug delivery, weaken unwanted inflammation, or enhance the required immune response. The design of nanoparticles with sequences that enhance or weaken the immune response allows them to be used as vaccine adjuvants in the first variant, as well as immunoquiescent drug delivery in the second one [144,145].

A series of publications devoted to the use of RNA nanoparticles demonstrates the positive experience of using nanoparticles with appropriate ligands for the treatment of cancer. The studies indicate that RNA nanoparticles are characterized by chemical and mechanical stability and can be assembled in vitro from modular blocks, which simplifies their quality control (see reviews by Haque et al.; Rossetti et al.) [68,146].

The main approach used to develop nanoparticles is in vitro synthesis, primarily using reverse transcription methods. To date, a wide variety of self-assembling structures have been obtained in vitro, such as squares, mosaics, filaments, cubic frameworks, and polyhedra [132].

However, the high cost of synthesizing large amounts of RNA nanoparticles using reverse transcription, as well as the need to study their properties and stability within living cells, encourages research on nanoparticle synthesis approaches in vivo [147].

In vivo production of nucleic acid-based nanoparticles is a serious problem. This idea was originally expressed by Seeman in 1997 [148]. However, it was not until 2004 that an octahedron was enzymatically assembled from a long ssDNA and several short auxiliary oligonucleotides [149]. In 2011, it was possible to synthesize RNA molecules in *E. coli*, which formed spatial structures necessary for carrying out chemical reactions [150].

In subsequent years, several studies investigated the synthesis of ssDNA in bacterial cells and their in vitro folding into specified nanostructures [151,152]. Research on the development of new strategies for the production of RNA nanoparticles in vivo continues, but it is still limited to *E. coli* [59].

The study of the opportunity to synthesize RNA nanoparticles in eukaryotic organisms is an important stage in the development of nanotechnology. On the one hand, this can contribute to improving the technology for producing RNA nanoparticles in vivo. On the other, it will allow us to approach the study of the properties of RNA nanoparticles and their functioning in eukaryotic cells.

Among the possible eukaryotic organisms for RNA nanoparticle production, the most interesting is the baker’s yeast *Saccharomyces cerevisiae*, which is one of the most popular model organisms in molecular biology and genetics. This is due to the combination of simplicity of cultivation, with the possibility of performing complex genetic manipulations, as well as the conservation of many key processes in yeast and mammalian cells. One of the distinguished features of *S. cerevisiae* is the absence of an RNA interference (RNAi) system, which was lost during evolution.

As a result, stable endogenous RNAs can be found in yeast cells, and this was demonstrated in the example of double-stranded RNA viruses of the genus Totivirus (L-A viruses) and single-stranded RNA viruses of the Narnavirus genus, belonging to the Narnaviridae family [153]. Yeast is also seen as a promising system for the synthesis and delivery of siRNAs [154,155].

RNA has also been an interesting molecule in drug design, including the first FDA-approved RNAi therapy for hereditary transthyretin-mediated amyloidosis in 2018 [156] and the mRNA vaccines against SARS-CoV-2 [157].

For the synthesis of RNA aptamers or RNA nanoparticles, yeast strains with the T7 RNA polymerase structural gene integrated into the genome can be used [156,158]. Transformation of such strains with plasmids containing the required nucleotide sequence under the control of the T7 promoter will provide the synthesis of the RNA of interest [159]. A significant advantage of using eukaryotic organisms for the production of aptamers and RNA nanoparticles in comparison with bacteria is also the absence of endotoxins in eukaryotes, which simplifies the procedure for purifying the compounds of interest and preparing them for use in medicine [160].

## 8. Conclusions

Recent advances in the production of modified RNA aptamers and the improvement of their folding methods increase the efficiency and reliability of their production. Due to their unique properties—high affinity and selectivity of binding of the target molecule—aptamers are widely used in various fields of biological and medical sciences. They can be used to detect various target molecules and inhibit their activity and also to create sensitive and specific biosensors. Aptamers can be considered as promising candidates for the targeted delivery of drugs to certain cells and tissues [161].

The creation of fluorogenic RNA aptamers has facilitated the localization of target molecules in the cell, monitoring the transcriptional activity of the cell, studying the regulation of transcription, evaluating the activity of regulatory proteins, and assessing the level of low molecular weight metabolites. Fluorogenic aptamers can be used to diagnose diseases, localize tumors, and identify pathologically relevant proteins [74].

The development of RNA nanotechnology has paved the way for developing RNA nanoparticles with controlled structure, size, and favorable chemical and thermodynamic properties. RNA nanoparticles can be used as a means of targeted delivery of various drugs, including miRNAs, siRNAs [162]. Integration of RNA nanotechnology and CRISPR-Cas9 technology with the aim of possible genome editing also seems promising [163].

For the widespread use of RNA nanoparticles, it is necessary to improve the methods of their preparation. Until now, RNA nanoparticles have mainly been produced in vitro, relying on chemical or enzymatic synthesis. This requires the development of special separation methods. In vivo synthesis of RNA nanoparticles is an important aspect of RNA nanotechnology, because it will facilitate not only the obtaining of the nanoparticles but also the study of their properties and stability inside the cells. Some progress has been made in this area using *E. coli* cells [59]. However, the real success of RNA nanotechnology will be the synthesis of functional RNA nanoparticles in eukaryotic cells. Most likely, further efforts of researchers will be aimed at studying and developing eukaryotic organisms—producers of aptamers and RNA nanoparticles.

Adherence to good laboratory practice (GLP), good manufacturing practice (GMP), the development of standardized protocols for production and use of aptamers and nanoparticles, and the concerted efforts of scientists, industrial partners, clinicians, and other stakeholders are the prerequisites for the realization of RNA/DNA nanotechnology in clinical practice [164].

## Data Availability

Not applicable.

## Sample Availability

Samples of the compounds are available from the authors.
